# Editorial: Disease-modifying targets and strategies for Alzheimer's disease and Parkinson's disease

**DOI:** 10.3389/fnagi.2023.1247256

**Published:** 2023-07-14

**Authors:** Xiaoya Gao

**Affiliations:** ^1^Department of Pediatric Neurology, Zhujiang Hospital, Southern Medical University, Guangzhou, Guangdong, China; ^2^Department of Neurology, Zhujiang Hospital, Southern Medical University, Guangzhou, Guangdong, China

**Keywords:** Alzheimer's disease, Parkinson's disease, disease modification, neuroprotection, novel targets, neurodegeneration

Alzheimer's Disease (AD) and Parkinson's Disease (PD) (Bloem et al., 2021) are two of the most common neurodegenerative diseases in the world, affecting large numbers of elderly population. AD is characterized by a progressive decline in cognitive and behavioral abilities (Tank et al., 2022), while PD is typically characterized by motor symptoms (such as slowness of movement or tremor) and non-motor symptoms (such as hyposmia, sleep disorders and autonomic dysfunction) (Kalia and Lang, 2015). These symptoms significantly reduce the quality of life for patients. Unfortunately, there is no cure for AD and PD at present, and treatments focus on relieving symptoms rather than slowing the progression of the disease (Ma et al.; Michel et al., 2016). Therefore, there is an urgent need for new disease-modifying targets or strategies for AD and PD.

The following studies were dedicated to discovering new disease-modifying targets and developing new therapeutic strategies for AD and PD, as well as exploring the critical pathways and effects on patients. We believe that the new information obtained from these studies can be used to develop successful therapies that have the potential to delay and even reverse the onset and progression of AD and PD.

“*Synthetic circular RNA (circRNA) as a therapeutic strategy for Alzheimer's disease (AD)*” (Zhao et al., 2022). Synthetic circRNAs have recently been identified as a potential therapeutic strategy for AD. For example, ciRS-7 has been found to be downregulated in AD, which leads to an up-regulation of free miRNA-7 in the hippocampus and a consequent decrease in autophagic proteins such as ubiquitin protein ligase A. This hinders the degradation of amyloid-beta (Aβ) peptides. Zhao et al. (2022) proposed synthetic circRNAs as a potential treatment for AD and other neurodegenerative diseases.

“*Isoniazid improves cognitive performance, clears A*β *plaques and protects dendritic synapses in APP/PS1 transgenic mice*” (Chen et al.). Chen et al. tested the effects of INH on APP/PS1 transgenic mouse models of AD, and found that after receiving oral INH (45 mg/kg/d) for 14 days, the cognitive function of APP/PS1 mice was improved. In addition, INH reduced Aβ plaques in the hippocampus and cortex, and resulted in lower levels of free Aβ in the brain homogenates, cerebrospinal fluid, and serum. These results suggest that INH could be an effective drug for AD.

“*Moxibustion improves hypothalamus AQP4 polarization in APP/PS1 mice: evidence from spatial transcriptomics*” (Liu et al.). Studies have shown that moxibustion can improve the learning and memory abilities of those with AD. To further investigate this, spatial transcriptomics was used to examine the expression and polarization of Aquaporin-4 (AQP4) around the blood brain barrier in wild type mice, APP/PS1 mice, and APP/PS1 mice that had been treated with moxibustion. The results showed that moxibustion could restore cognitive function in APP/PS1 mice, likely due to an increase in the polarization of AQP4 in the hypothalamus, which is likely caused by increased mitochondrial energy supply. These pathways could be promising targets for modifying the disease.

“*Late-LTP magnitude, but not A*β *levels and Amyloid pathology, is associated with behavioral performance in a rat knock-in model of Alzheimer disease*” (Yesiltepe et al.). It is widely agreed that APP mutations can lead to the formation and accumulation of Aβ, which in turn can cause early-onset familial Alzheimer's disease. This is caused by the sequential cleavage of APP by β- and γ-secretases. Studies have demonstrated that the Swedish mutation causes deficits in higher order brain function and a heightened production of Aβ in 14-month-old App-Swedish (Apps) knock-in rats. However, there is no evidence of AD-associated amyloid and neuroinflammatory pathology in the APPs/s rats. To further investigate this, Yesiltepe et al. studied Long-term potentiation (LTP) and found that Apps/s control females showed an increase in LTP and this was correlated to behavioral deficits. The results suggest that pathways related to LTP dysfunction could be potential disease-modifying targets for AD.

“*Effects of preventive interventions on neuroimaging biomarkers in subjects at-risk to develop Alzheimer's disease: a systematic review*” (Perus et al.). This systematic review sought to examine the global and regional effects of preventive interventions (including physical exercise, nutrition, cognitive training or multidomain interventions) on brain biomarkers, particularly imaging biomarkers. It was highlighted that it is essential to select Alzheimer's patients based on their stage of the disease and risk factors before any interventions are carried out, and that frontal regions, which are known to be affected by neurodegeneration, may be a critical factor in the intervention process. In summary, preventive interventions may be an effective way to reduce brain degeneration in those at high risk of AD.

“*A nomogram based on iron metabolism can help identify apathy in patients with Parkinson's disease*” (Li et al.). Studies have been showing a link between abnormal iron metabolism and apathy in PD. Li et al. conducted a study to examine the clinical features and iron metabolism of PD patients with apathy and created a nomogram to predict apathy in PD. Their results showed that in the apathetic group, serum transferrin and total iron binding capacity (TIBC) levels were lower and negatively correlated with Apathy Scale (AS) scores in male PD patients, indicating that abnormal iron metabolism may be involved in apathy in PD, especially in males, and could be a potential therapeutic target. The nomogram generated by the study had good discrimination and calibration, with a high consistency index (0.799, 95% confidence interval = 0.732–0.865), based on the combination of age, sex, serum iron concentration, TIBC and Hamilton Depression Rating Scale scores.

“*Identification and immune characteristics of molecular subtypes related to protein glycosylation in Alzheimer's disease*” (Ma et al.). Protein glycosylation is involved in the development of AD, but systematic analysis of the relationship between protein glycosylation-related genes (PGRGs) and immune system process is still lacking in AD. Eight PGRGs (*SLC7A11, S100A10, LGALS3, CD55, CHST14, GSTP1, DYNC1H1, and ADAMTS8*) were identified as differentially expressed between the training set and control samples. Using a machine learning approach, AD was divided into two subtypes based on the expression of core PGRGs, with the high protein glycosylation subtype exhibiting higher levels of neuroinflammation. The expression pattern of PGRGs and immune infiltration varied between the two subtypes. A diagnostic nomogram model was constructed, with SERPINA3 identified as the key biomarker for distinguishing between the two subtypes and playing a critical role in the pathological progression of AD. Protein glycosylation and related pathways may be potential therapeutic targets for AD, and riluzole and sulfasalazine may have clinical utility and warrant further investigation.

Researchers have been working on developing novel cellular models to identify potential modifying targets for AD and PD. However, there is no single “perfect” model for the two diseases. In “*A primary rodent triculture model to investigate the role of glia-neuron crosstalk in regulation of neuronal activity*” (Phadke et al.), a new cellular model was designed. The optimized model includes neurons, astrocytes and microglia in a specially designed medium. Compared to standard cocultures, exogenous microglia were added in the model. This functional triculture model could be used for inspecting glia-neuron interactions, thereby studying neuroinflammation, hyperexcitability and relevant cellular crosstalk *in vitro*. So far, the model has been proved to be quantitative and reproducible and to aid in drug development.

In summary, these articles provide several novel targets and strategies for modifying AD and PD. These targets are involved in immunity, inflammation, mitochondria, energy metabolism and other related pathways. The authors also proposed some practical and reliable models for further exploration of disease-modifying treatments.

## Author contributions

XG is responsible for writing and revised the original draft.
